# Mitigating banding artifacts in balanced steady‐state free precession using parallel transmission in a single acquisition

**DOI:** 10.1002/mrm.70106

**Published:** 2025-10-12

**Authors:** Chia‐Yin Wu, Jin Jin, Markus Barth, Martijn A. Cloos

**Affiliations:** ^1^ Centre for Advanced Imaging, The University of Queensland St. Lucia Queensland Australia; ^2^ ARC Training Centre for Innovation in Biomedical Imaging Technology University of Queensland St. Lucia Queensland Australia; ^3^ School of Electrical Engineering and Computer Science University of Queensland St. Lucia Queensland Australia; ^4^ Imaging Centre of Excellence University of Glasgow Glasgow UK; ^5^ Siemens Healthineers Pty Ltd Brisbane Queensland Australia; ^6^ Donders Centre for Cognitive Neuroimaging Radboud University Nijmegen Netherlands

**Keywords:** banding artifact, balanced SSFP (bSSFP), parallel transmission (pTx), ultra‐high field

## Abstract

**Purpose:**

Using parallel transmission (pTx) in ultra‐high field MRI allows efficient radiofrequency pulse designs that can produce uniform excitations. Here, we demonstrate that pTx can simultaneously mitigate $B_{1}^{+}$ non‐uniformity and off‐resonance artifacts in balanced steady‐state free precession sequences at 7 T.

**Theory and Methods:**

The standard hard excitation pulses of the balanced steady‐state free precession sequence were replaced with two pTx pulses, played out in alternating repetition times. Based on the k_T_‐point trajectory, each pulse was designed to produce spatially varying phase distributions that compensate for off‐resonance phase variations accumulated over one repetition time duration, while also mitigating $B_{1}^{+}$ non‐uniformities. Depending on the local ΔB_0_, the different steady‐state signals formed were distributed over two aliased images with a half field‐of‐view shift relative to one another. Using slice‐GRAPPA, these images were disentangled and recombined to produce artifact‐free images.

**Results:**

Simulations validated the concept showing that a pair of strategically designed pTx pulses can impose a favorable distribution of spatially varying steady‐state. As a proof of principle, experiments performed demonstrated the technical feasibility of the concept in a phantom in the presence of induced phase variations and in vivo.

**Conclusion:**

With a tailored pair of pTx pulses, it was possible to concurrently mitigate $B_{1}^{+}$ and ΔB_0_ non‐uniformities in a thin slab through the brain in a single acquisition. Although further work is needed to extend the coverage and robustness of this method before being adopted more widely, it highlights the potential of utilizing pTx capabilities beyond $B_{1}^{+}$ correction.

## 1 INTRODUCTION

The balanced steady‐state free precession (bSSFP) sequence is one of the most efficient acquisition strategies in terms of signal‐to‐noise ratio (SNR) per unit acquisition time.^1^ The signal is formed by a large magnetization that is maintained by a steady‐state oscillation. Minor dephasing effects due to off‐resonance and T_2_' are mitigated by a spin echo–like refocusing effect at the echo time, where TE = TR/2. However, it is still sensitive to inhomogeneities in the magnetic field (ΔB_0_). As the accumulated phase per TR approaches 180°, the desired steady‐state breaks down. As a result, the signal disappears and manifests as banding artifacts in the image.

Many techniques have been suggested to combat banding artifacts. For applications targeting a relatively small region of interest (ROI) at low fields (e.g., 1.5 T and 3 T), such as cardiac MR, a frequency scout^2^ can be applied to optimize the frequency for the ROI. This method, however, is limited to ROIs containing little ΔB_0_ variation. Alternatively, the periodic banding artifacts observed in bSSFP can be spatially shifted by manipulating the increments of the phase cycle between subsequent radiofrequency (RF) pulses.^3^ Combining multiple acquisitions with varying RF phase cycling patterns can mitigate the appearance of banding artifacts in the final image. For example, a combination of two acquisitions, one with a 0º–180° and one with 0º–0° phase cycling, can be combined to fill in each other's signal voids.^4^, ^5^, ^6^ However, the use of multiple acquisitions with different phase cycling schemes compromises the time efficiency of bSSFP and increases susceptibility to motion.

Parallel transmission (pTx) provides extra flexibility to shape the transmit field ($B_{1}^{+}$). It uses multiple transmitters, each independently driven by a separate RF amplifier that allows the $B_{1}^{+}$ to be manipulated in space and time. In its simplest form, referred to as RF shimming, a tailored fixed combination of relative phase and amplitudes is set to improve the $B_{1}^{+}$. RF shimming can improve the $B_{1}^{+}$ locally, but it is often difficult to homogenize $B_{1}^{+}$ throughout the brain at 7 T and higher fields.^7^, ^8^, ^9^ Varying the weight and phase combination of the transmitters throughout the RF pulse enables acceleration in transmit k‐space. Such pulses can implement almost any arbitrary magnetization pattern. In particular, short pulses can be used that create uniform excitation (spokes, k_T_‐points).^9^, ^10^ For example, non‐selective k_T_‐points pulses can be used to generate uniform excitations by targeting a sparse distribution of discrete k‐space locations with short rectangular RF pulses.^9^

Here we demonstrate the design of a pair of tailored pTx pulses that simultaneously compensate for $B_{1}^{+}$ non‐uniformities and banding artifacts in bSSFP. This approach forces the magnetization into spatially variant steady‐state oscillations specifically designed to counteract the signal dephasing caused by ΔB_0_. This approach captures signal from two spatially varying bSSFP phase cycling patterns in one acquisition simultaneously, mitigating both $B_{1}^{+}$ and banding artifacts even though the underlying $B_{1}^{+}$ and ΔB_0_ are heterogeneous. The theory is described, followed by proof of principle experiments in phantoms and in the human brain at 7 T.

## 2 THEORY

**FIGURE 1:**
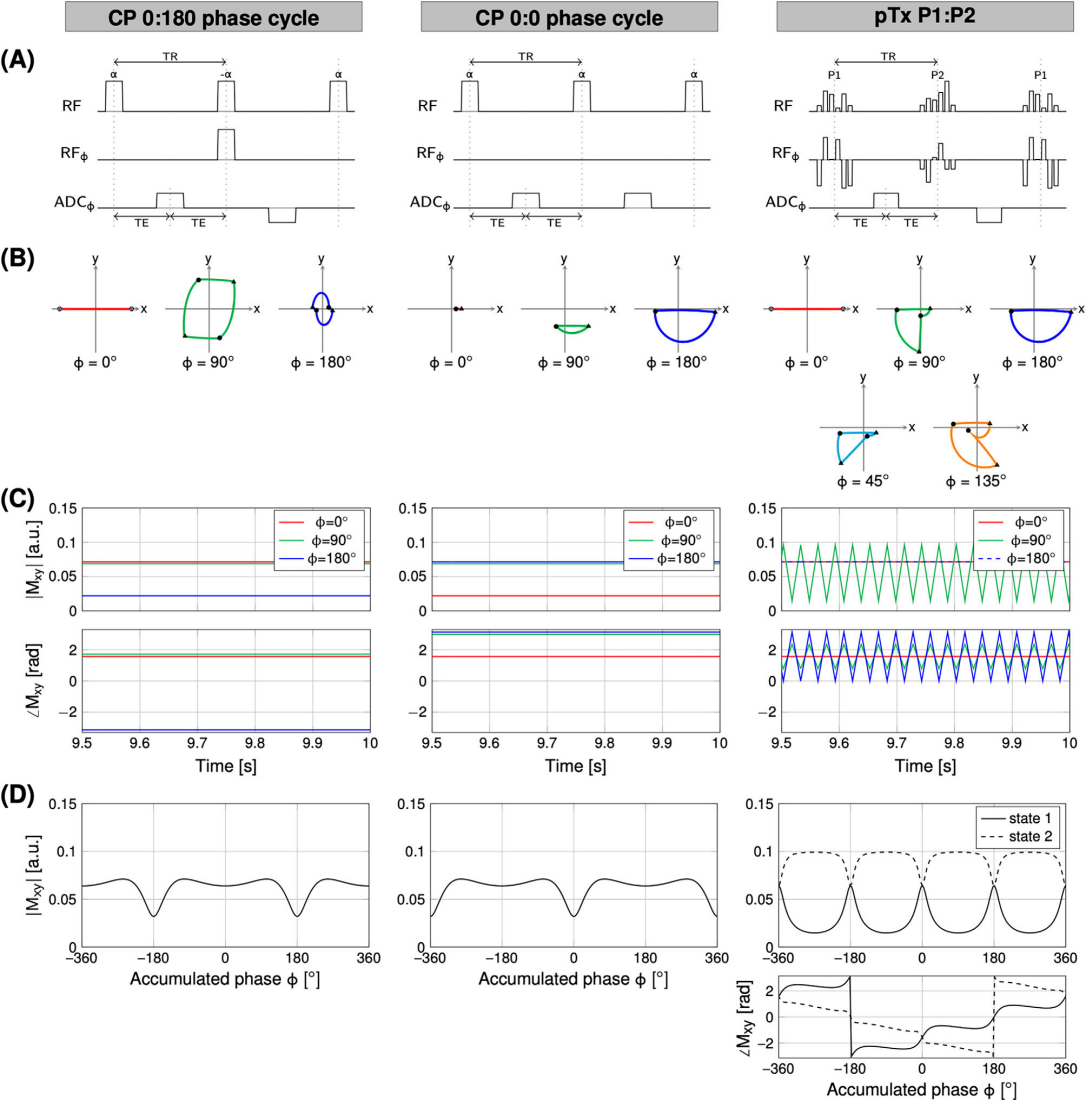
(A) Balanced SSFP sequence diagram showing RF magnitude, RF phase, and analog‐to‐digital converter (ADC) phase for circularly polarized (CP)–mode 0:180 phase cycling (*left*), CP‐mode 0:0 phase cycling (*middle*), and parallel‐transmission (pTx)–mode P1:P2 pulse pair (*right*) implementations. (B) The oscillating motion of magnetization in steady‐state per TR projected on the xy‐plane of the rotating frame for cumulative phase *φ* of 0°, 90°, and 180° per TR. For the P1:P2 pTx solution, the trajectory of the magnetization is also shown for 45° and 135° of phase accumulation per TR. The duration of the RF pulse applied is indicated by the circle (*start*) and triangle (*stop*) markers. (C) Spin magnetization evolution once steady‐state is reached for 0°, 90°, and 180° phase accumulation. Phase coherence with respect to the ADC is consistent for all TRs and is adhered to for both CP‐mode phase cycling patterns, whereas magnitude oscillation and phase incoherence is observed using pTx P1:P2 in every other TR for off‐resonant frequencies. (D) Signal amplitude as a function of φ. Note that the pTx P1:P2 solution creates two states with complementary dynamics.

The bSSFP signal is formed by alternating ±*α* excitation pulses (Figure 1A, 0:180 phase cycling). This creates a refocusing mechanism which mitigates dephasing accumulated during one TR. However, alternating ±*α* pulses fail to compensate for phase accumulation (*φ*) approaching 180°. As a result, the desired steady‐state breaks down and the signal disappears (Figure 1B, 0:180 phase cycling).

Figure 1D shows the signal amplitude as a function φ. The periodic dips in the signal indicate when banding artifacts will form. These banding artifacts can be shifted in space by altering the phase cycling pattern. For example, a 0:0 phase cycling pattern shifts the bands 90° (Figure 1, center column). The desired steady‐state is now driven for off‐resonance spins (*φ* = 180°) and breaks down for on‐resonance spins (*φ* = 0°). Although each of the individual images will still have banding artifacts, the two can be combined to obtain a single image with reduced banding artifacts.^4^, ^5^, ^6^

Here we show that, using pTx, a pair of two tailored RF pulses can be designed that enforce spatially varying phase cycling patterns in one single bSSFP acquisition to simultaneously mitigate banding and $B_{1}^{+}$ artifacts (Figure 1, right column). To accomplish this, a spatially varying phase difference of γΔB_0_TR is enforced between the target phase of the pTx pulse pair (P1 and P2). This compensates for the spatially varying phase accumulated due to off‐resonance over one TR. When played in the bSSFP sequence, the phase imposed by the pTx P1:P2 pulses drives all, on‐resonance and off‐resonance spins, into a favorable steady‐state. As a result, spins will follow a 0:180‐*γ*ΔB_0_TR RF phase cycling pattern depending on the off‐resonance at the corresponding spatial location. As such, spatially on‐resonance spins will experience 0:180° RF phase cycling, whereas the spins accumulating close to 180° dephasing will experience 0:0° RF phase cycling (Figure 1B).

**FIGURE 2:**
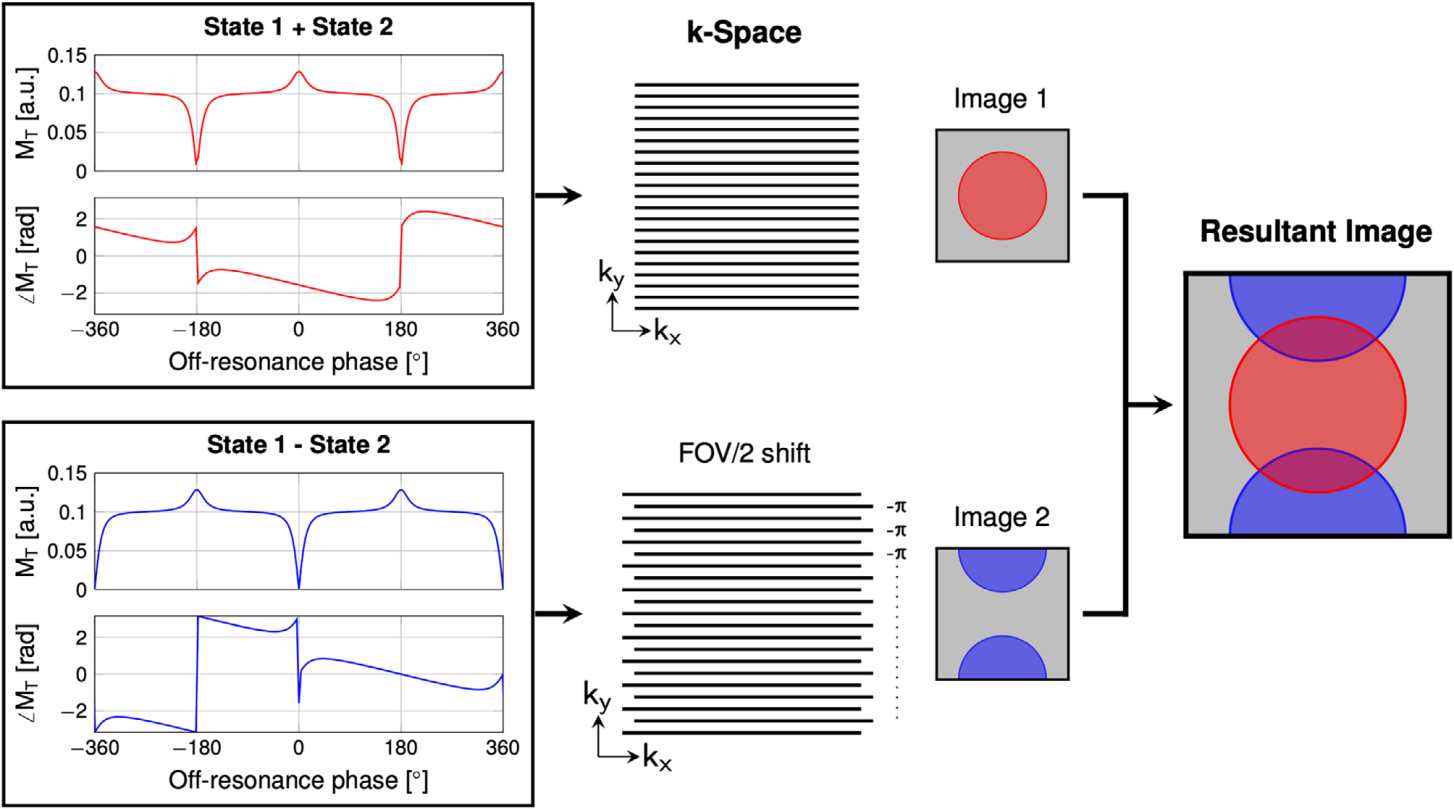
The underlying relationship between spin states and k‐space influenced by the pTx pulse pair (P1:P2). The observed signal in Image 1 and Image 2 is generated by the addition or subtraction of the State 1 and State 2 complex signal shown in Figure 1. For consistency, a 0:180 analog‐to‐digital converter (ADC) phase cycling pattern is set as reference. This is a representation of the produced k‐space data respective to the ADC and resultant image showing superimposed images generated by the respective combination of State 1 and State 2.

Spatially varying phase cycling also implies that, in every other TR, a subpopulation of spins will be 180° out of phase relative to the analog‐to‐digital converter (ADC). This can be interpreted as having two images shifted by half field of view (FOV) as shown in Figure 2. Image 1 contains a superposition of the signal from the two states (Figure 2, top graph). Image 2 contains the difference between the signal from the two states (Figure 2, bottom graph). The resultant image with two virtual slices superimposed onto one another mimics the effect of a blipped controlled aliasing in parallel imaging (CAIPI) approach (i.e., RF phase shift^11^), and therefore can be separated and recombined using slice‐GRAPPA.^12^

## 3 METHODS

### 3.1 Experimental setup

All data were collected using a 7 T whole‐body research scanner (Magnetom 7 T Plus; Siemens Healthineers, Erlangen, Germany) equipped with an 8 × 2 kW parallel transmit system, an 8‐transmit/32‐receive head coil (Nova Medical, Wilmington, MA, USA) and gradient model SC72 (max slew rate = 200 mT/m/ms, *G*_max_ = 70 mT/m). A homemade 3% agar phantom (NaCl = 2.5 g/L, T_1_ = 2.5 s, T_2_ = 50 ms) was used for experimental validation. Calibration data were acquired as previously shown.^13^ In summary, a SA2RAGE^14^ (flip‐angle [FA] = 6°, TR = 2400 ms, TE = 0.95 ms, TD1 and TD2 = 5 ms, 1700 ms, matrix = 48 × 64 × 64, resolution = 4 × 4 × 4 mm^3^, *T*_acq_ = 115 s) was acquired in circularly polarized (CP)‐mode. In combination, a two‐dimensional (2D) interleaved gradient‐recalled echo (GRE) (FA = 5°, TR = 300 ms, TE = 3 ms, matrix = 48 × 64 × 64, resolution = 4 × 4 × 4 mm^3^, GRAPPA = 3, *T*_acq_ = 64 s) was acquired by selectively pulsing on each individual transmit channel and in CP‐mode to calculate a full set of absolute $B_{1}^{+}$ maps for each transmit channel. Additionally, three‐dimensional GRE images with two different echoes (FA = 8°, TR = 5 ms, TE1 and TE2 = 1.7 ms, 2 ms, matrix = 96 × 128 × 128, resolution = 2 × 2 × 2 mm^3^, *T*_acq_ = 63 s) were acquired for ΔB_0_ mapping. A linear B_0_ distortion was applied by adjusting the shim parameter in the phase direction to create an off‐resonance variation between ±60 Hz. As a result, dephasing is larger than ±180° for the corresponding TR parameter set in the bSSFP sequence. The required slice‐GRAPPA calibration lines used to disentangle the two superimposed images created by alternating between the two pTx pulses were acquired separately using a spoiled GRE sequence (FA = 5°, TR = 5 ms, TE = 1.7 ms, matrix = 112 × 128 × 128, resolution = 1.5 × 1.5 × 1.5 mm^3^) in CP‐mode.

### 3.2 PTx dual‐pulse design

**FIGURE 3:**
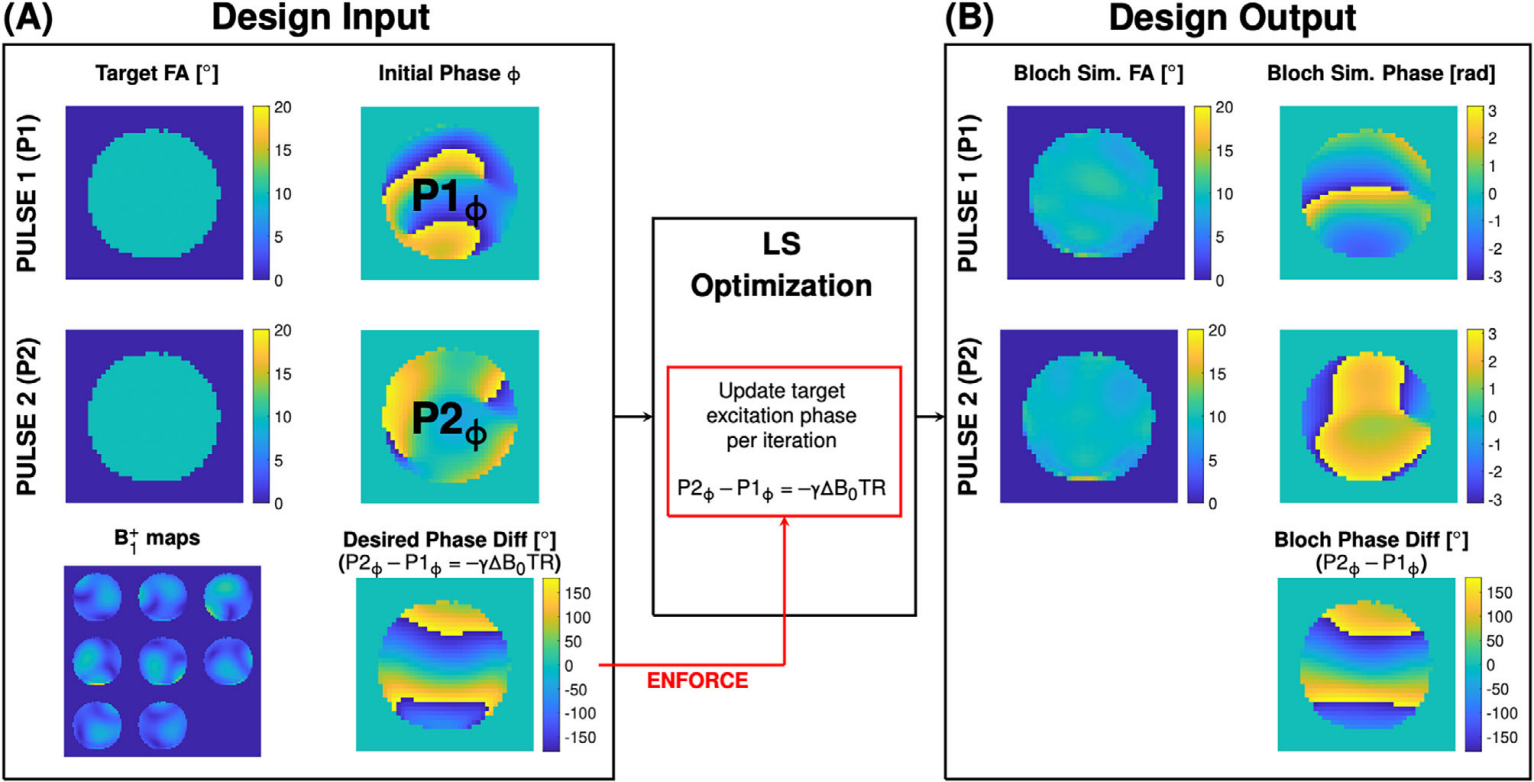
Overview of the magnitude least squares pulse design workflow showing required design inputs (A) and outputs (B). Full Bloch simulations were performed with the designed pulses to evaluate pulse fidelity. The initial target phase of the two pTx pulses were set to the CP–mode phase ±(*γ*Δ*B*_0_TR)/2. The CP‐mode‐like background phase was iteratively relaxed, but the −*γ*Δ*B*_0_TR difference remained enforced. FA, flip‐angle; LS, least squares; TR, repetition time.

A conventional non‐selective bSSFP sequence was modified to allow pairs of pTx pulses to be played in alternating TRs (i.e., P1:P2) or the same pTx pulse for every TR (e.g., P1:P1). A pair of k_T_‐points pulses were designed simultaneously (Figure 3) using the spatial domain method with a magnitude least‐squares approach.^9^, ^15^, ^16^ The target amplitude was set to 1° FA during design and linearly scaled to the desired FA in application. The initial target phase maps were set to the phase of the CP coil mode with a ± (*γ*ΔB_0_TR)/2 phase shift superimposed, for P1 and P2, respectively. Subsequently, the absolute phase constrained is relaxed through iterative redesign using the average phase produced by the two pulses in the previous iteration and reinforcing the ±(*γ*ΔB_0_TR)/2 difference (Figure S1). Individual subpulse durations were optimized such that the RF amplitudes were limited to $B_{1}^{+}$ of 7.5 uT. This generated a set of k_T_‐points with variable subpulse duration that can effectively reduce the overall pulse duration while balancing RF power.^9^ A predefined symmetric k_T_‐point trajectory ensured applied gradient blips were balanced within one TR (Figure S2). The source code and sample data set files are available at github (https://github.com/yinwu26/dualPTxPulse).

### 3.3 Acquisition

The bSSFP (FA = 15°, TR = 15 ms, TE = 7.5 ms, matrix = 112 × 128 × 128, resolution = 1.5 × 1.5 × 1.5 mm^3^, *T*_acq_ = 215 s) images were acquired using the CP‐mode and pTx‐mode with a conventional single optimized pTx pulse, both with and without phase cycling. The pair of two tailored pTx pulses were designed separately to the single optimized pTx pulse. To verify the phase difference between P1 and P2 pulses, bSSFP images were acquired without alternating between pulses (i.e., P1:P1 and P2:P2). The phase difference between these images was compared with the target phase difference. The comprehensive solution was tested by alternating between pulses, referred to as the P1:P2 configuration. All P1:P2 measurements used 0:180 ADC phase cycling.

The proposed pTx P1:P2 solution was evaluated under in vivo conditions by scanning the brain of a healthy volunteer. Before the scan, the subject provided written informed consent of the project ethics, which was approved by the local human research ethics committee in accordance with national guidelines. All calibration data and bSSFP sequences were performed using the same parameters as those used for in vitro.

### 3.4 Image reconstruction

Here we show two approaches of separating the two states and forming a final image. First, slice‐GRAPPA (kernel size 5 × 5) was implemented to disentangle the two superimposed images (Image 1 and Image 2) produced by the P1:P2 configuration. A final image was obtained using sum‐of‐squares (SoS) method by combining signals from both images after removing the FOV/2 phase shift. Alternatively, GRAPPA (kernel size 5 × 4) can be used to reconstruct two separate images using only the odd or even phase encoded lines in k‐space. The image reconstructed using odd k‐space lines contain State 1 signal, whereas the reconstruction using even k‐space lines only contain State 2 signal. The complex addition and subtraction, as explained in Section 2, was applied to produce the two images corresponding to the Image 1 and Image 2 signals.

The g‐factor and SNR corresponding to different acceleration factors were evaluated using a pseudo‐multiple‐replica approach.^17^ A dedicated noise scan was acquired to calculate the noise covariance matrix. A comparison between pTx P1:P1 (combined 0:0 and 0:180) and pTx P1:P2 was evaluated for acceleration factors of two (*R* = 2) and four (*R* = 4) such that the total scan time was matched. For the pTx P1:P1 method, the acceleration factor was applied to each of the two complementary phase cycling acquisitions. Undersampling was applied in the phase‐encoding direction of k‐space.

## 4 RESULTS

**FIGURE 4:**
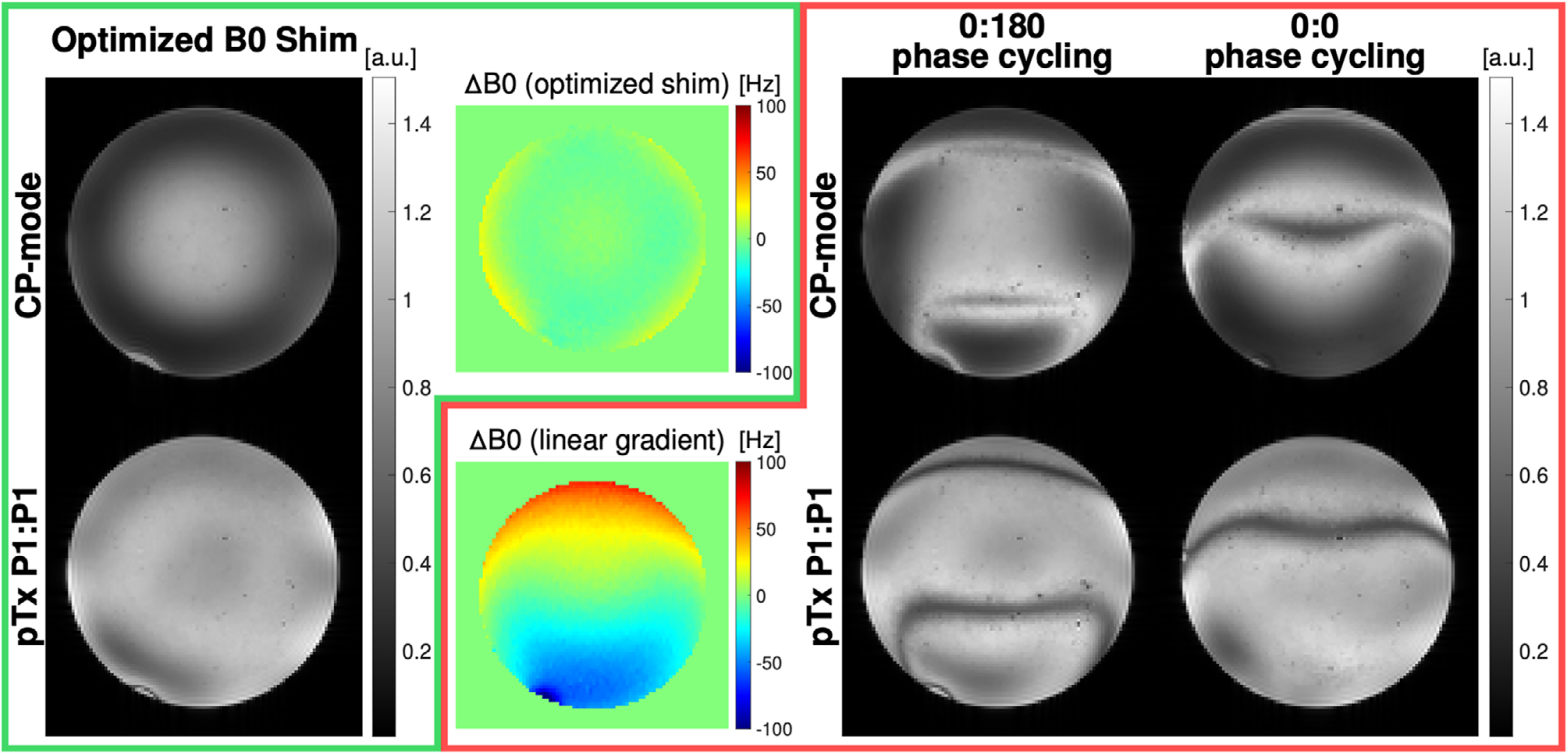
Comparison of the bSSFP images acquired using the CP‐mode and conventional single optimized pTx k_T_‐points pulse with different phase cycling schemes (0:180 and 0:0). Each colored box shows the resulting bSSFP image corresponding to one of the two ΔB_0_ distributions. The green box shows bSSFP images acquired with minimal ΔB_0_ variation, and the red box shows bSSFP images acquired with a ΔB_0_ gradient ranging between ±60 Hz.

Figure 4 shows the bSSFP images acquired with and without phase cycling in the CP‐mode and using a single optimized k_T_‐points pulse (P1:P1). When ΔB_0_ is small enough to avoid exceeding 180° phase accumulation over one TR (green box), there are no banding artifacts, whereas the $B_{1}^{+}$ artifacts (CP‐mode) can be mitigated using pTx. When a large B_0_ gradient is introduced (red box), the CP‐mode demonstrated both $B_{1}^{+}$ and banding artifacts. When using a single tailored pTx pulse, $B_{1}^{+}$ artifacts are mitigated, although the banding artifacts remain. Using different phase cycling schemes can move the bands but not mitigate them in a single acquisition.

**FIGURE 5:**
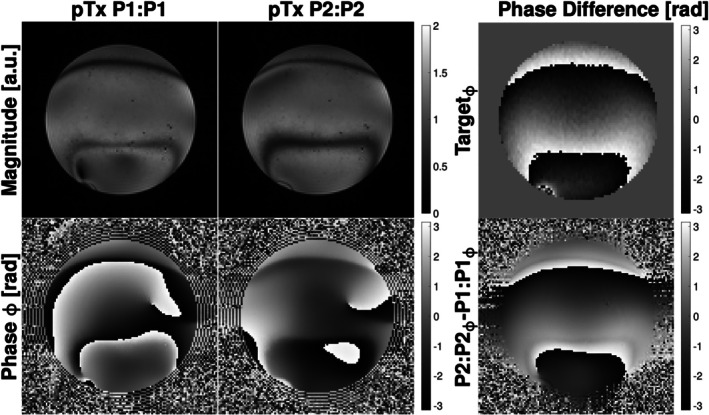
Magnitude and phase images acquired in pTx‐mode with P1:P1 and P2:P2 pulses acquired separately in the bSSFP sequence. Both acquisitions were acquired with 0:180 ADC phase cycling. The column on the right shows the target phase difference used in the pulse design and the phase difference generated by the two pTx pulses (P2:P2‐P1:P1).

Figure 5 shows the experimental verification of our proposed pTx pulse pair played out independently in the bSSFP sequence (P1:P1 and P2:P2) under an induced linear ΔB_0_ variation between ±60 Hz. Given the exception to the banding artifacts observed, each of the pulses produced a uniform excitation. The third column in Figure 5 shows that the phase difference generated by the pair of these pulses matches the target phase difference needed to compensate for the ΔB_0_ gradient seen in Figure 4 (red box).

**FIGURE 6:**
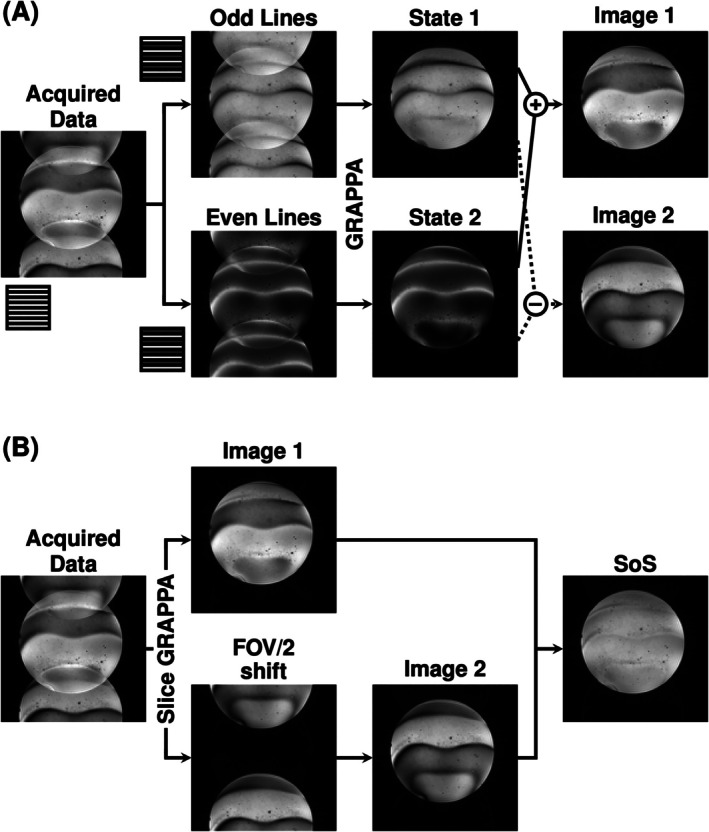
(A) Reconstruction of the P1:P2 measurement (*Column 1*) by sorting even and odd lines into two separate images (*Column 2*). Column 3 shows the even and odd sorted images reconstructed using GRAPPA. The final column shows the images obtained by complex addition or subtraction of States 1 and 2. Theory indicates that Images 1 and 2 should match the P1:P2 slice‐GRAPPA reconstruction. (B) Experimental validation of the bSSFP sequence using P1:P2 pulse pair in pTx‐mode. Full reconstruction workflow using slice‐GRAPPA to separate Image 1 and Image 2. Final resultant image of combining Image 1 and Image 2 after FOV adjustment.

To verify the signal dynamics described in Section 2, the superimposed images in the measured P1:P2 data was reconstructed using only odd or even lines (Figure 6A). The rightmost column shows the complex summation of the two images obtained by adding or subtracting these two states.

Figure 6B shows the use of slice‐GRAPPA to separate the superimposed states in the P1:P2 measurement. These two images match the combination of signal states seen in Figure 6A. Combining the two images results in an image with minimal excitation nonuniformity and significantly mitigated banding artifacts.

**FIGURE 7:**
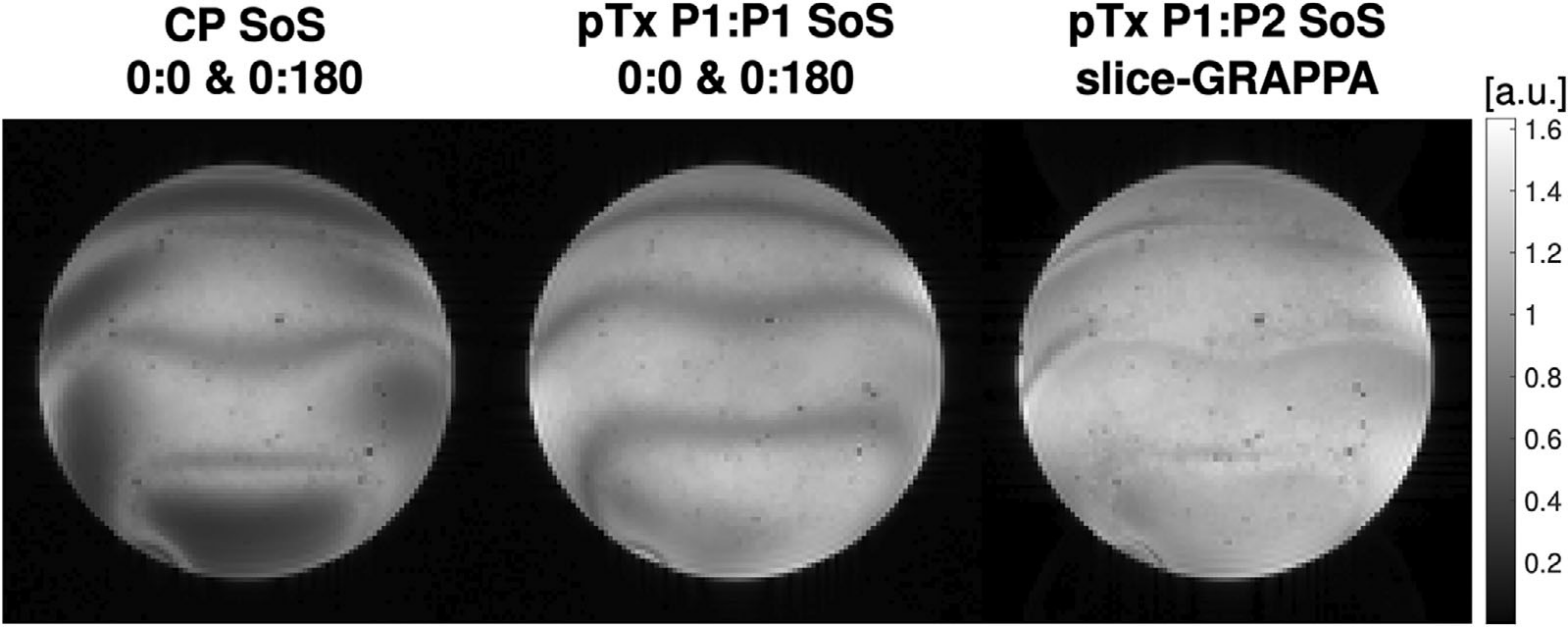
Comparison of composite bSSFP images generated in different modes. *Left to right*: Standard hard pulse excitations in CP‐mode, single optimized tailored pTx pulse (P1:P1), and the proposed pTx P1:P2 pulse solution. The CP and P1:P1 solutions combined two separate acquisitions (0:180 and 0:0 phase cycling). The P1:P2 solution was obtained using a single acquisition. All acquisitions were acquired with the linear B_0_ gradient (Figure 4, red box) imposed.

Figure 7 shows the resultant bSSFP images for the different pulse configurations. The left and middle show the composite image of both phase cycling schemes combined using the CP‐mode and a single optimized pTx pulse (P1:P1), respectively. The signal drop in peripheral regions seen in the CP‐mode with a coefficient of variation (CoV) of 0.33, is improved by using pTx pulses (CoV = 0.21). Compared with the uncombined images in Figure 4, the banding artifacts are reduced. The third image shows the combination of slice‐GRAPPA images produced by the P1:P2 pTx pulse pair with further mitigation of the banding artifacts (CoV = 0.19). The signal extracted in the two images is complementary, thus simultaneously addressing $B_{1}^{+}$ and ΔB_0_ non‐uniformity in a single acquisition.

### 4.1 In vivo

**FIGURE 8:**
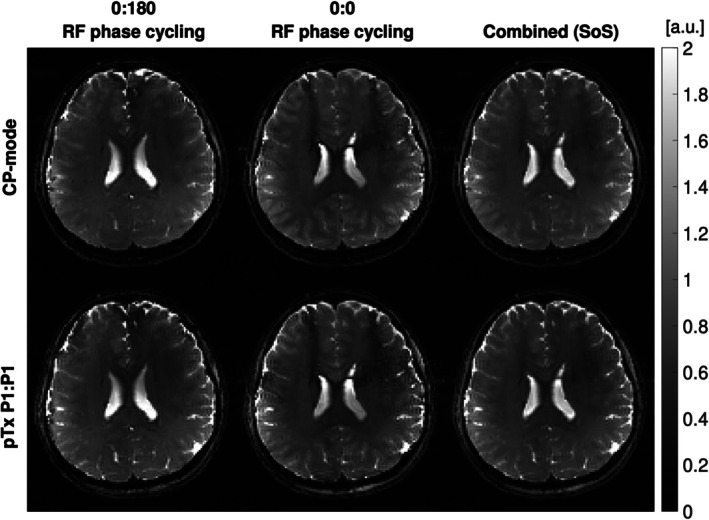
The bSSFP images acquired in vivo using CP‐mode (*top row*) and conventional single optimized pTx P1:P1 (*bottom row*) solution. *Left to right*: 0:180, 0:0 phase cycling, and the combined image. All images were acquired without parallel imaging.

Figure 8 shows axial slices through the brain obtained using the CP‐mode and P1:P1 solutions, where pulse duration of P1 = 2.13 ms. As expected, the ΔB_0_ variation results in distinct banding artifacts. In both pulse configurations, the 0:0 and 0:180 phase cycling schemes produced complementary banding artifacts. Although a visual inspection of the SoS images appears to indicate relatively similar homogeneity, the k_T_‐point solution improved the excitation (9% non‐uniformity) compared with the CP‐mode (44% non‐uniformity).

**FIGURE 9:**
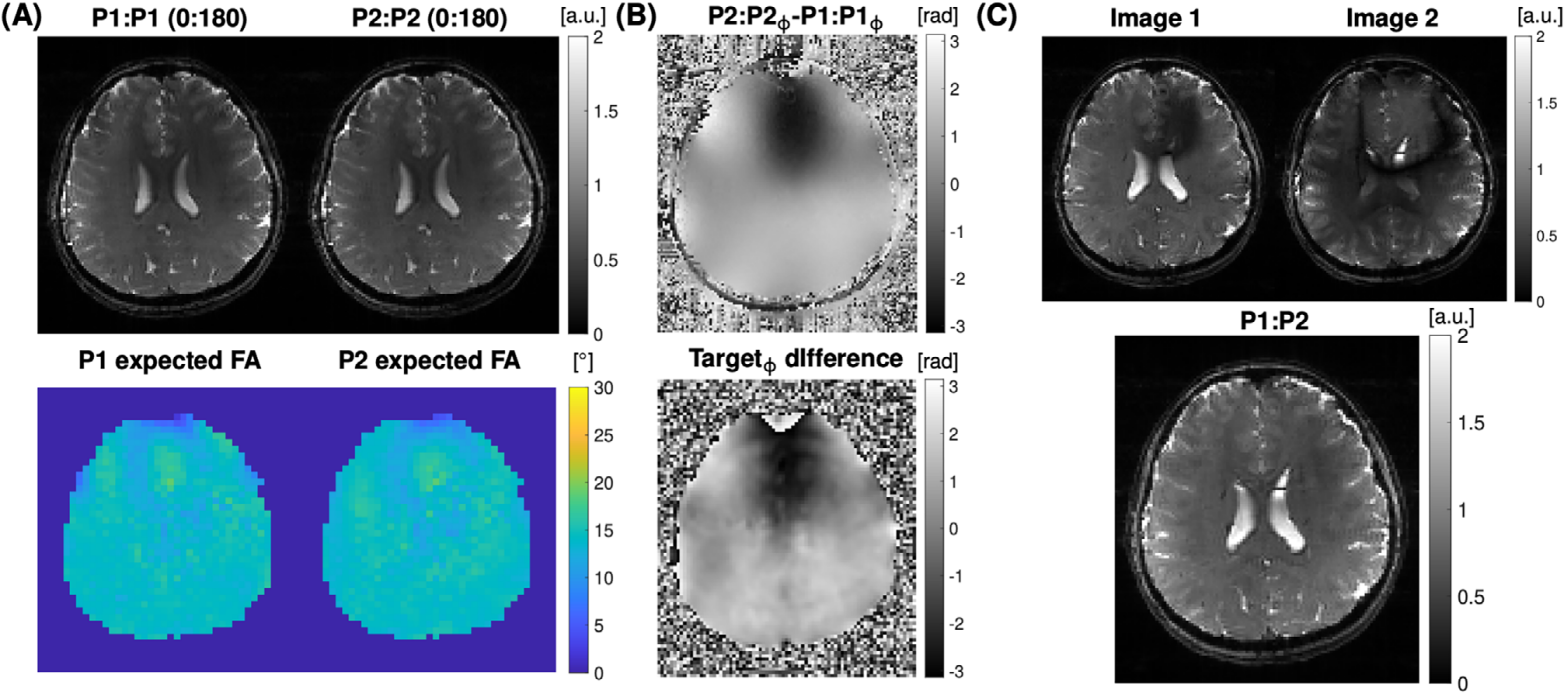
Evaluation of pTx P1:P2 optimized on an axial slice of the brain. (A) images of bSSFP acquired using P1:P1 and P2:P2. The expected excitation profile with a 15° flip‐angle using Bloch simulation is shown for P1 and P2 in the bottom panel. (B) Verification of the measured phase difference to the target phase difference. (C) Reconstructed image using slice‐GRAPPA of bSSFP sequence using pTx P1:P2 solution. Top panel shows the complementing Image 1 and Image 2 extracted using slice‐GRAPPA, after field‐of‐view adjustment of Image 2. All images were acquired without parallel imaging.

Figure 9 shows the results obtained using the P1:P2 solution. The pulse design converged to a 10% non‐uniformity of the magnitude $B_{1}^{+}$ fields for both P1 (pulse duration = 3.62 ms) and P2 (pulse duration = 3.93 ms) (Figure 9A). The phase difference produced by the P1 and P2 pulses closely matched the target phase pattern (Figure 9B). Using slice‐GRAPPA, the individual images show the expected complementary signal distribution (Figure 9C). The combination of slice‐GRAPPA images produced a banding artifact mitigated bSSFP image (Figure 9C, bottom) comparable to images obtained using two separate measurements (Figure 8, third column).

**FIGURE 10:**
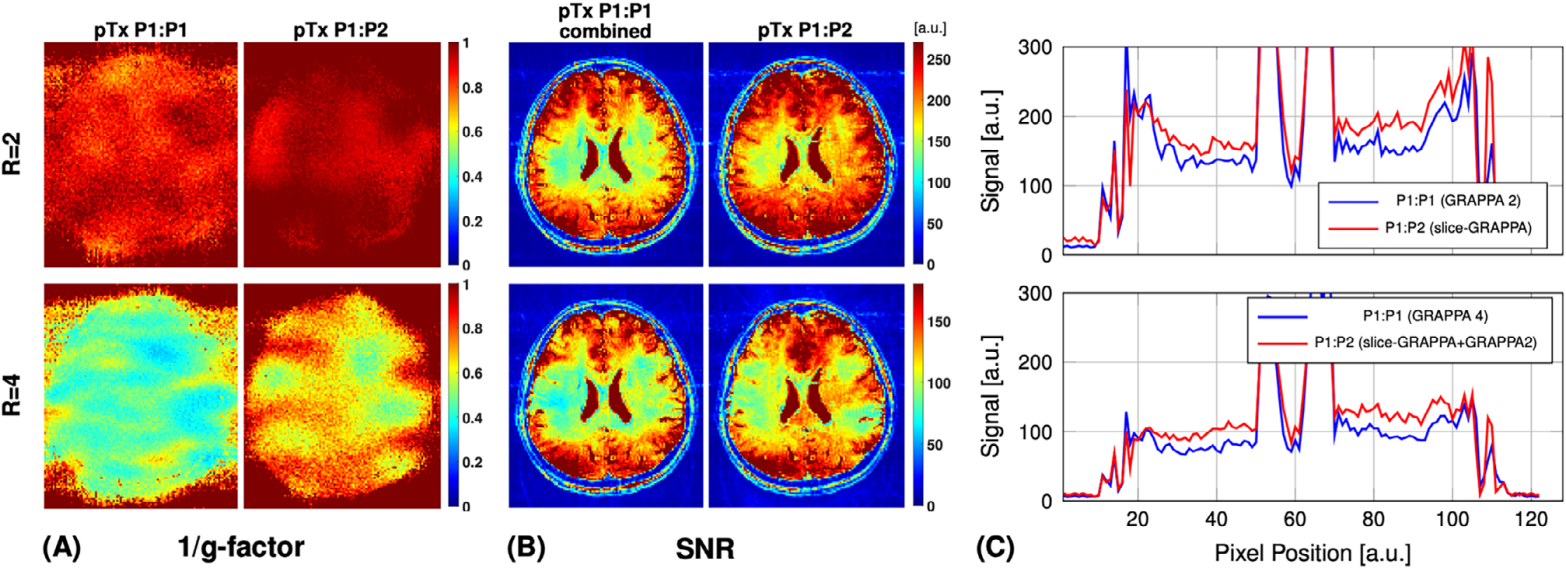
(A) Retained SNR (1/g‐factor). (B) SNR maps estimated using pseudo‐multiple replicas for a total acceleration factor of two (*R* = 2) and four (*R* = 4). The maps in each row correspond to measurements with equal total scan time. For acquisitions using conventional single optimized pTx pulses (P1:P1), undersampling was applied to each of the two complementary phase cycling schemes. The left column of each panel shows the estimated pTx P1:P1 maps, and the second column shows the pTx P1:P2 maps. (C) Comparison of the mid‐horizontal cross‐sectional SNR signal profile for *R* = 2 (*top*) and *R* = 4 (*bottom*).

Figure 10A shows the retained SNR (1/g‐factor) maps. For both acceleration factors (*R* = 2 and *R* = 4), the pTx P1:P2 configuration showed superior performance with a lower noise‐amplification penalty characterized by the g‐factor. Figure 10B shows the estimated SNR maps of the combined (0:0 + 0:180) pTx P1:P1 image and pTx P1:P2 image reconstructed with different acceleration factors. The slice‐GRAPPA reconstruction of the proposed P1:P2 acquisition showed improved SNR performance compared with the combined pTx P1:P1 reconstructed with GRAPPA = 2 (Figure 10, top row). The pTx P1:P2 solution in combination with GRAPPA = 2 in‐plane, giving a total acceleration of *R* = 4, also outperformed the (0:0 + 0:180) pTx P1:P1 strategy using GRAPPA = 4 (Figure 10, bottom row). Note that in each of these comparisons, the total scan time was equal.

## 5 DISCUSSION

This work presents a pTx pulse design specifically tailored to simultaneously mitigate $B_{1}^{+}$ inhomogeneity and off‐resonance effects in bSSFP. A unique feature of our proposed solution is the use of a pTx pulse pair that drives spatially tailored steady‐state patterns to compensate for spatially variable off‐resonance frequencies.

For the standard bSSFP, all gradient pulses are balanced between two consecutive excitation pulses. To ensure this balance was adhered to, a symmetric pTx pulse trajectory was used. A standard pTx solution that optimizes flip‐angle only in the brain at 7 T generally only need to visit a small number of locations (e.g., 3 k_T_‐points) in the center of transmit k‐space.^18^, ^19^ Effectively, with fewer k_T_‐points, the TR may be shortened, making the bSSFP sequence more robust to off‐resonance effects (Figure S3). However, banding artifacts remain at isocenter and in inferior slices of the brain. Mitigating these artifacts still requires combining at least two separately acquired images with 0:180 and 0:0 phase cycling. However, off‐resonance variations vary much more rapidly than $B_{1}^{+}$. Therefore, when both $B_{1}^{+}$ and ΔB_0_ are simultaneously considered, a greater number of subpulses that cover more locations in transmit k‐space are required to target both low and high spatial frequencies. In the phantom experiment, a high correlation between the target and experimental phase‐difference pattern was achieved with relative ease—presumably because the induced ΔB_0_ variation was relatively linear. However, in vivo, the ΔB_0_ distribution often varies non‐linearly and more rapidly than $B_{1}^{+}$. Consequently, it was much more difficult to design P1:P2 pulse pairs for in vivo use. As a proof of principle, we decided to focus on a single axial slice through the brain. Although neighboring slices containing similar ΔB_0_ and $B_{1}^{+}$ variations still worked reasonably well, further work is needed to extend the method to whole brain coverage, including challenging areas such as near the ear canals.

Because the ΔB_0_ varies more quickly than $B_{1}^{+}$, the P1:P2 strategy is most likely more dependent on the k‐space trajectory. Moreover, in our approach it is the TR that dominates the effective ΔB_0_ induced phase change. Considering that the TR is much longer than the RF pulse duration considered in conventional k_T_‐points pulse designs, the optimization of pulses for our bSSFP P1:P2 strategy is much more challenging. Although we did not yet attempt to optimize the transmit k‐space locations, it may be expected that transmit k‐space locations need to target higher spatial frequencies to compensate for fast‐changing off‐resonance effects throughout the brain. Alternative transmit k‐space trajectories could be considered, such as a spiral trajectory.^19^, ^20^ In general, there are many different optimization techniques that could be used to design a set of P1:P2 solutions. The current design implementation is relatively rigid where the phase accumulated in each TR is split 50/50 between the two pulses. However, in principle, the phase correction could be distributed arbitrarily between the pulses. For example, we also optimized the pair of pulses by alternatingly redesigning one pulse based on the last design outcome of the other pulse. This relaxes the constraint that enforces each pulse to take care of half the difference phase target. Alternatively, it also allows the design to start with a phase noise target. Unlike the CP‐mode phase, white phase noise offers unbiased initial targets. Although the theoretical performance was better, we also found the power distributed between the two pulses to become extremely imbalanced. Potentially the power distribution could be rebalanced by dynamically adjusting regularization parameter throughout the process.^21^ If local specific absorption rate (SAR) information is available, then optimization using virtual observation points may be best,^22^ potentially “hopping” between local SAR distributions with each pulse.^23^

As predicted by simulations, FOV/2 shift aliasing occurred when using our pTx P1:P2 pulse solution, due to the spatially variable phase cycling patterns. Interestingly, the signal response of the odd‐only or even‐only echoes (Figure 1D: P1:P2) may be perceived as two distinct steady‐states, whereby the effective TR is doubled. However, unlike doubling the TR in a standard bSSFP acquisition with a fixed phase cycling scheme, the spins still “see” an RF pulse for all TRs with the pTx P1:P2 implementation. There, P1 and P2 produce different phase distributions such that the effective phase cycling scheme now varies point by point. Most important is that the banding patterns produced by these states remain complimentary.

Combining different phase cycling schemes generally work better at higher FAs, as the bands become wider and vary more smoothly. However, the systems current conservative SAR estimation prevented the use of short TR times and high FAs with reasonable pulse durations (Table S1). Furthermore, with higher number of subpulses, lengthening the RF pulse durations become impractical. Longer pulses would lead to longer TR, which induces more severe banding artifacts. Future work would explore a combination of improved SAR monitoring, local SAR constrains, and SAR hopping to enable larger FAs.

Our proposed pTx P1:P2 approach captures multiple spatially varying steady‐state patterns in one acquisition. To match the scan time of our proposed pTx P1:P2 approach, the standard multi‐acquisition bSSFP approach that acquires two complementary phase cycling schemes would require parallel imaging with an acceleration factor of *R* = 2 applied to each of the two acquisitions independently. Subsequently, an acceleration factor of *R* = 4 in each of the two P1:P1 acquisitions matches the total scan time of the pTx P1:P2 method with additional GRAPPA = 2 in‐plane applied. Our proposed dual pulse pTx solution inherently aliases the alternating steady‐state signal evolutions, generating two complementary signals with FOV/2 shift. This can be thought of as two separate images, or “virtual slices,” superimposed onto each other with a FOV/2 shift. Viewed as a sensitivity‐encoding reconstruction problem, the shifted slice has “virtual” coil sensitivities associated with it, whereby they are shifted in space by FOV/2. These virtual receive coil sensitivities, “virtual coil,” help reduce the g‐factor penalty. Conversely, conventional sensitivity‐encoding/GRAPPA reconstructions with in‐plane undersampling strive to unfold one single image using the receive sensitivity profiles from the target image alone. Additionally, because all lines in k‐space are acquired for the P1:P2 approach, there is no reduction in SNR due to undersampling. As a result, our P1:P2 approach was able to outperform the standard bSSFP acquisition with *R* = 2 acceleration. Furthermore, the P1:P2 solution can still be combined with different variations of GRAPPA combinations to enable higher acceleration factors. An example of applying in‐plane GRAPPA in combination with the P1:P2 solution to give *R* = 4 acceleration was demonstrated. However, other variations of GRAPPA implementation (e.g., 2D CAIPI)^11^, ^24^ may further reduce g‐factor penalties for both the standard and proposed methods. Future in‐depth studies are required to explore the vast array of parallel imaging approaches to clarify the most SNR efficient approach.

This proof of principle study focused on the human brain at 7 T. Looking into the future, an implementation of the proposed technique could also find clinical application in cardiac MRI at 1.5 T and 3 T. Due to the challenges imposed by cardiac motion, acquisition speed is a coveted commodity. Collecting separate images with different phase cycling patterns to compensate for banding artifacts can lead to motion related artifacts. The opportunity to capture two spatially varied steady‐state patterns in one acquisition would eliminate the motion between complementary images.

## 6 CONCLUSION

We have shown that pTx can be used to simultaneously mitigate banding and $B_{1}^{+}$ artifacts in bSSFP scans, highlighting exciting new potential‐use cases for pTx. Nevertheless, further work is needed to extend this proof of principle to whole brain coverage. Furthermore, the shown principle can be extended to other field strengths and body regions where bSSFP is of high relevance, such as cardiac imaging at 3 T.

## CONFLICT OF INTEREST

Jin Jin is employed by Siemens Healthineers Pty Ltd, Brisbane, Queensland, Australia.

## Supporting information


**FIGURE S1.** Flow diagram of the reinforced target phase modification in the variable exchange method for least squares (LS) optimization. An initial circularly polarized (CP)–mode target phase is used. For each subsequent iteration, the new target phase maps are set by the average phase produced by the two pulses (b_P1_, b_P2_) and reinforced with the ±(*γ*ΔB_0_TR)/2 difference.
**FIGURE S2.** Diagram of symmetric k_T_‐points pulse design with 13 subpulses (A) and centrally balanced gradients. (B) The color‐coded gradient blips correspond to a segment of the k‐space trajectory.
**FIGURE S3.** Bloch simulations of the standard 3D bSSFP sequence with two independent phase cycling schemes (0:180 and 0:0) and the resultant combined sum‐of‐squares (SoS) magnetization image. A single conventional 3k_T_‐points pTx pulse was optimized using the in vivo calibration data and replaced the standard hard excitation pulse in the bSSFP sequence. The target flip‐angle was 13°, and TR was 8 ms. For comparison, a TR of 15 ms was also simulated for the matched target slice of interest shown in the main manuscript (*top row*). The corresponding ΔB_0_ maps for simulated slice 1 cm in head direction from isocenter (H10) and at isocenter (iso).
**TABLE S1.** Relative specific absorption rate (SAR) and normalized root mean square error (NRMSE) for P1:P2 pulses designed for varying repetition times (TR) and flip‐angles (FA).
