# Supplementary material for: Mitigating banding artifacts in balanced steady‐state free precession using parallel transmission in a single acquisition
Source: Magn Reson Med. 2025 Oct 12;95(2):1017–29. doi: 10.1002/mrm.70106 (PMC12681311; doi:10.1002/mrm.70106)
Supplement: Supplementary file 1 — FIGURE S1. Flow diagram of the reinforced target phase modification in the variable exchange method for least squares (LS) optimization. An initial circularly polarized (CP)–mode target phase is used. For each subsequent iteration, the new target phase maps are set by the average phase produced by the two pulses (bP1, bP2) and reinforced with the ±(γΔB0TR)/2 difference. FIGURE S2. Diagram of symmetric kT‐points pulse design with 13 subpulses (A) and centrally balanced gradients. (B) The color‐coded gradient blips correspond to a segment of the k‐space trajectory. FIGURE S3. Bloch simulations of the standard 3D bSSFP sequence with two independent phase cycling schemes (0:180 and 0:0) and the resultant combined sum‐of‐squares (SoS) magnetization image. A single conventional 3kT‐points pTx pulse was optimized using the in vivo calibration data and replaced the standard hard excitation pulse in the bSSFP sequence. The target flip‐angle was 13°, and TR was 8 ms. For comparison, a TR of 15 ms was also simulated for the matched target slice of interest shown in the main manuscript (top row). The corresponding ΔB0 maps for simulated slice 1 cm in head direction from isocenter (H10) and at isocenter (iso). TABLE S1. Relative specific absorption rate (SAR) and normalized root mean square error (NRMSE) for P1:P2 pulses designed for varying repetition times (TR) and flip‐angles (FA). [file MRM-95-1017-s001.docx]

Supporting Information

Mitigating banding artifacts in bSSFP using parallel transmission in a single acquisition

Chia-Yin Wu^1,2,3,4^, Jin Jin^1,2,5^, Markus Barth^1,2,3^, Martijn A Cloos^1,2,6^

^1^Centre for Advanced Imaging, The University of Queensland, St Lucia, Australia

^2^ARC Training Centre for Innovation in Biomedical Imaging Technology, University of Queensland, St Lucia, Australia

^3^School of Electrical Engineering and Computer Science, University of Queensland, St Lucia, Australia

^4^Imaging Centre of Excellence, University of Glasgow, Glasgow, United Kingdom

^5^Siemens Healthineers Pty Ltd, Brisbane Australia

^6^Donders Centre for Cognitive Neuroimaging, Radboud University, Nijmegen, Netherlands

# Supporting Figure S1:


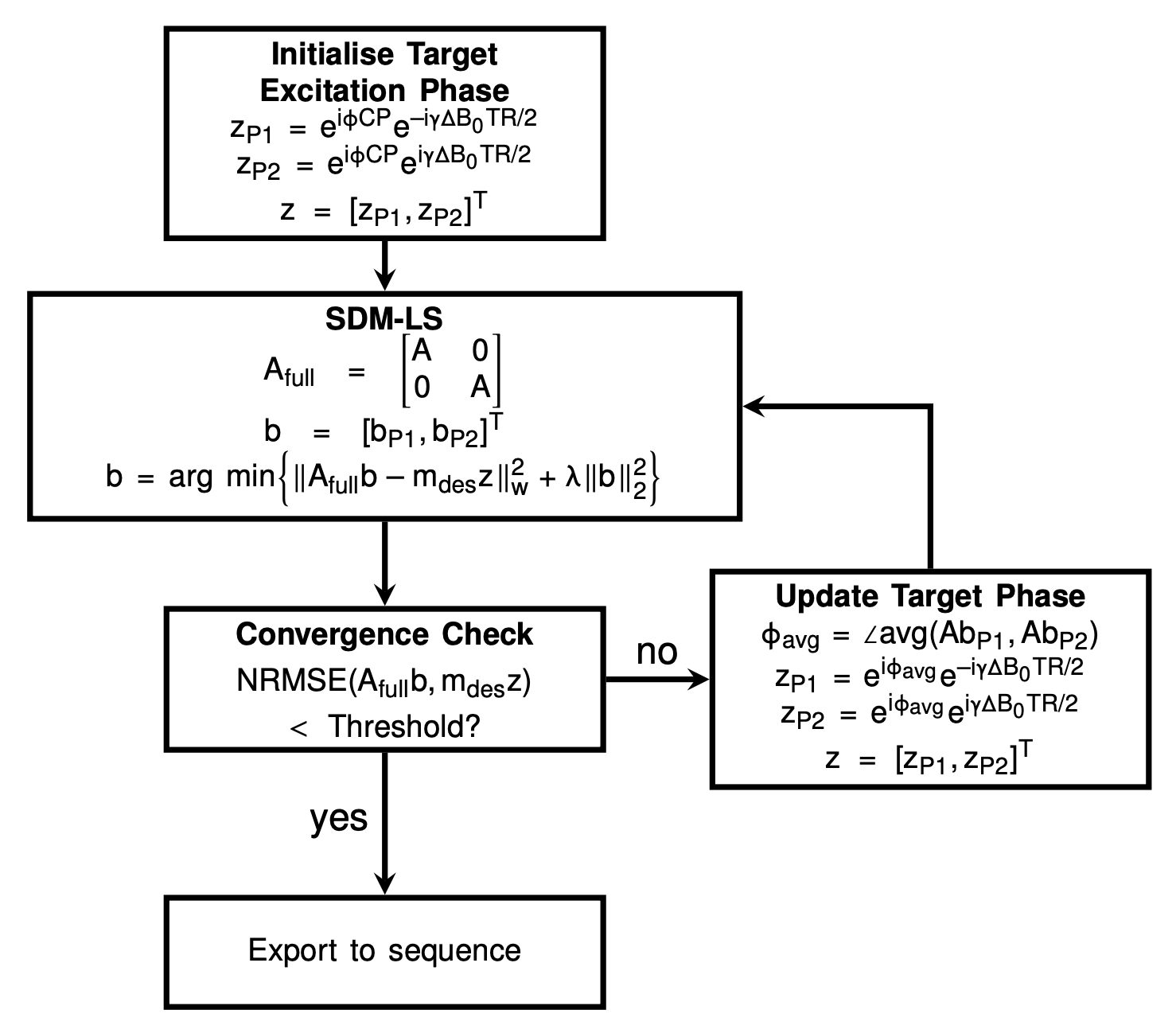


**Figure S1**: Flow diagram of the reinforced target phase modification in the variable exchange method for LS optimization. An initial CP-mode target phase is used. For each subsequent iteration, the new target phase maps are set by the average phase produced by the two pulses (b_P1_, b_P2_) and reinforced with the ±(γΔB_0_TR)/2 difference.

# Supporting Figure S2:


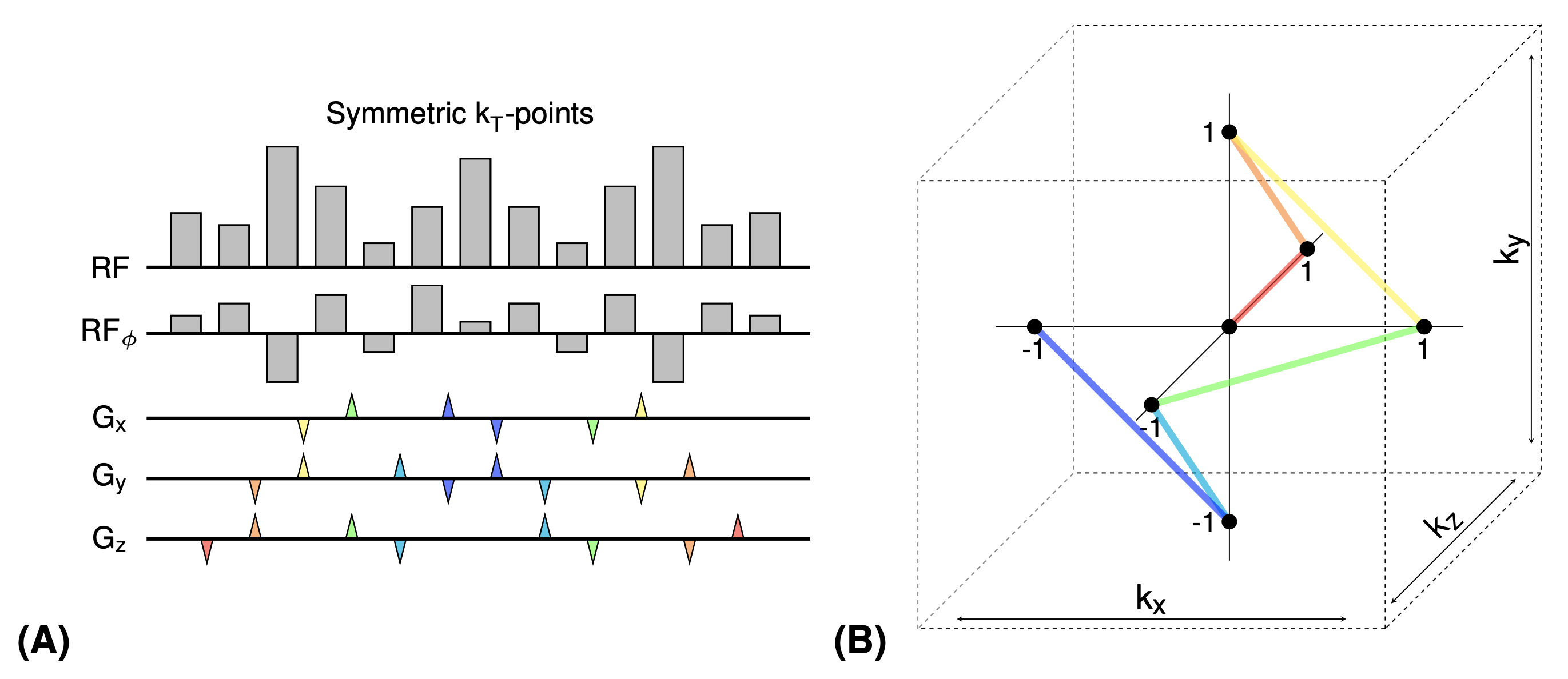


**Figure S2**: Diagram of symmetric k_T_-points pulse design with 13 sub-pulses (A) and centrally balanced gradients. The color-coded gradient blips correspond to a segment of the k-space trajectory (B).

# Supporting Figure S3:


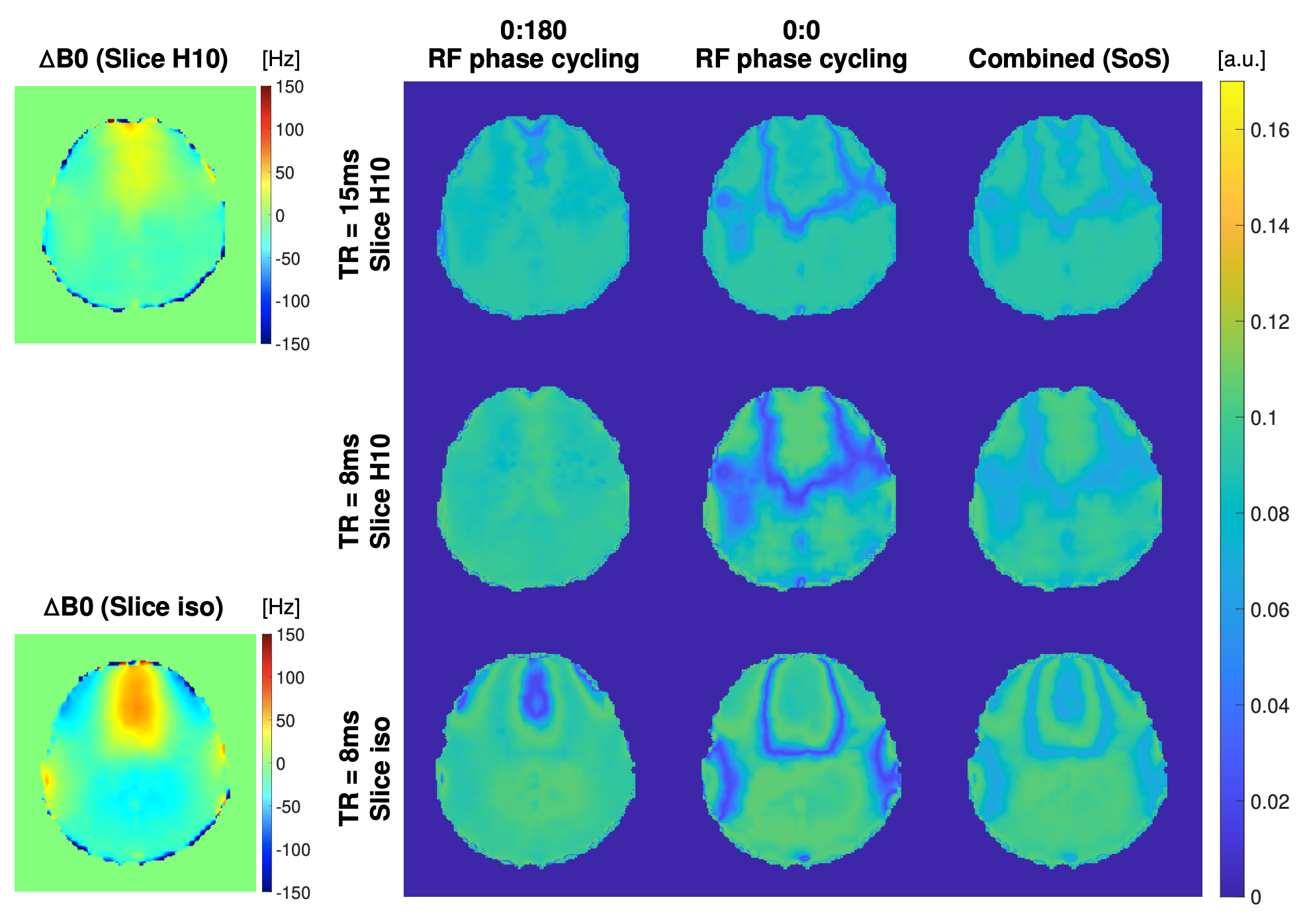


**Figure S3:** Bloch simulations of the standard 3D bSSFP sequence with two independent phase cycling schemes (0:180 and 0:0) and the resultant combined sum of squares (SoS) magnetization image. A single conventional 3k_T_-points pTx pulse was optimized using the in vivo calibration data and replaced the standard hard excitation pulse in the bSSFP sequence. The target flip-angle was 13 degrees and TR was 8ms. For comparison, a TR of 15ms was also simulated for the matched target slice of interest shown in the main manuscript (top row). The corresponding ΔB0 maps for simulated slice 1cm in head direction from isocentre (H10) and at isocentre (iso).

# Supporting Table S1:

|  | **TR=8ms** | | **TR=15ms** | | **TR=30ms** | |
| --- | --- | --- | --- | --- | --- | --- |
|  | **SAR %** | **NRMSE (P1/P2)** | **SAR %** | **NRMSE (P1/P2)** | **SAR %** | **NRMSE (P1/P2)** |
| **FA=10°** | 53 | 0.06/0.04 | 44 | 0.12/0.05 | 35 | 0.34/0.20 |
| **FA=15°** | 119 | 0.07/0.04 | 100 | 0.12/0.05 | 78 | 0.34/0.21 |
| **FA=30°** | 477 | 0.09/0.07 | 400 | 0.15/0.07 | 313 | 0.35/0.24 |
| **FA=45°** | 1073 | 0.14/0.12 | 900 | 0.19/0.12 | 705 | 0.38/0.29 |

**Table S1**: Relative SAR and normalized root mean square error (NRMSE) for P1:P2 pulses designed for varying TRs and flip-angles (FA).
